# Comparative analyses of DNA repeats and identification of a novel Fesreba centromeric element in fescues and ryegrasses

**DOI:** 10.1186/s12870-020-02495-0

**Published:** 2020-06-17

**Authors:** Jana Zwyrtková, Alžběta Němečková, Jana Čížková, Kateřina Holušová, Veronika Kapustová, Radim Svačina, David Kopecký, Bradley John Till, Jaroslav Doležel, Eva Hřibová

**Affiliations:** 1 Institute of Experimental Botany, Czech Academy of Sciences, Centre of the Region Haná for Biotechnological and Agricultural Research, Šlechtitelů 31, CZ-77900 Olomouc, Czech Republic; 2 Centro de Genómica Nutricional Agroacuícola, Las Heras 350, Temuco, Chile

**Keywords:** *Festuca*, *Lolium*, Illumina sequencing, Repetitive DNA, Centromere organization

## Abstract

**Background:**

Cultivated grasses are an important source of food for domestic animals worldwide. Increased knowledge of their genomes can speed up the development of new cultivars with better quality and greater resistance to biotic and abiotic stresses. The most widely grown grasses are tetraploid ryegrass species (*Lolium*) and diploid and hexaploid fescue species (*Festuca*). In this work, we characterized repetitive DNA sequences and their contribution to genome size in five fescue and two ryegrass species as well as one fescue and two ryegrass cultivars.

**Results:**

Partial genome sequences produced by Illumina sequencing technology were used for genome-wide comparative analyses with the RepeatExplorer pipeline. Retrotransposons were the most abundant repeat type in all seven grass species. The Athila element of the Ty3/gypsy family showed the most striking differences in copy number between fescues and ryegrasses. The sequence data enabled the assembly of the long terminal repeat (LTR) element Fesreba, which is highly enriched in centromeric and (peri)centromeric regions in all species. A combination of fluorescence in situ hybridization (FISH) with a probe specific to the Fesreba element and immunostaining with centromeric histone H3 (CENH3) antibody showed their co-localization and indicated a possible role of Fesreba in centromere function.

**Conclusions:**

Comparative repeatome analyses in a set of fescues and ryegrasses provided new insights into their genome organization and divergence, including the assembly of the LTR element Fesreba. A new LTR element Fesreba was identified and found in abundance in centromeric regions of the fescues and ryegrasses. It may play a role in the function of their centromeres.

## Background

**Fig. 1:**
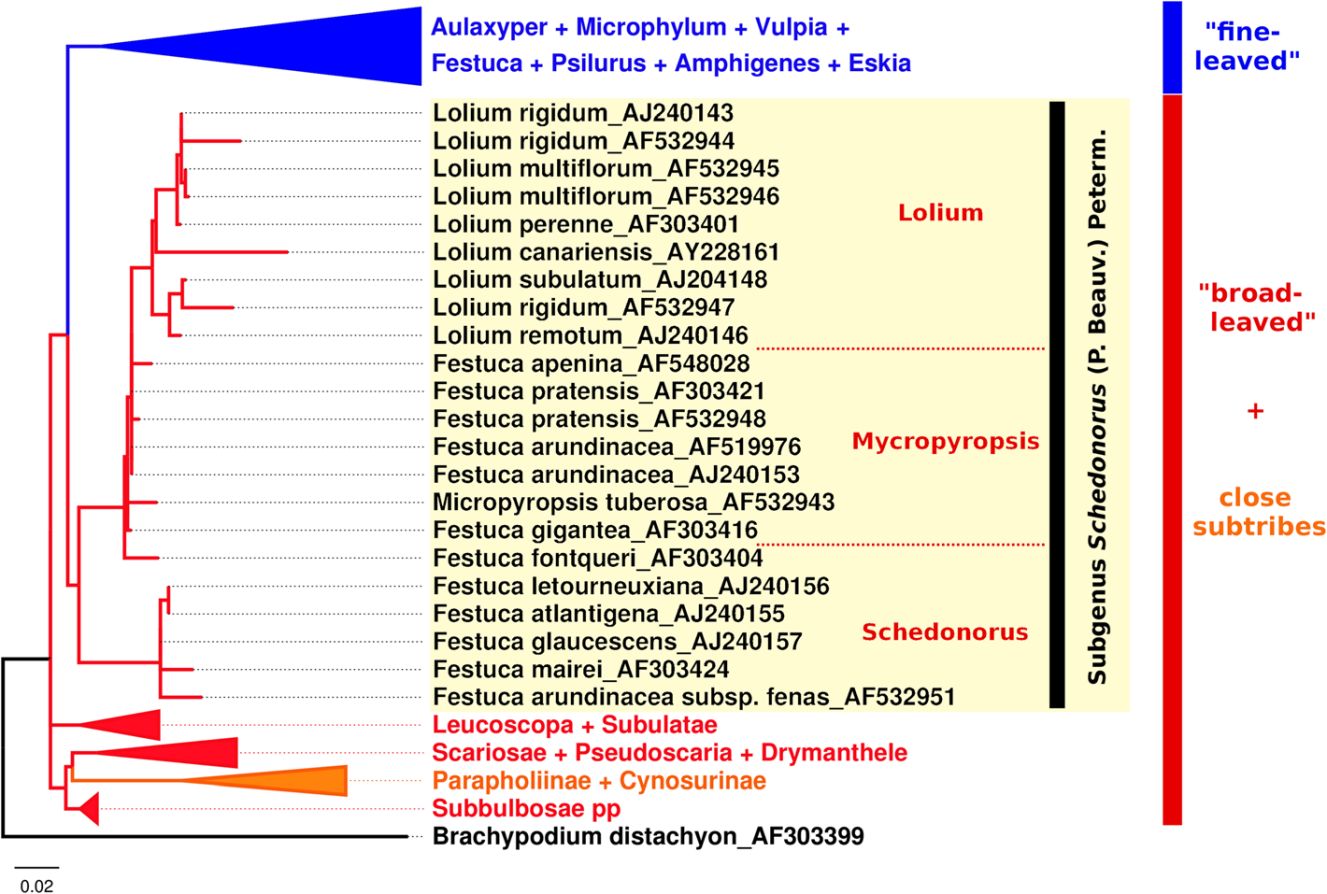
Phylogenetic tree of *Loliinae* subtribe. Phylogeny of subtribe Loliinae with *Brachypodium distachyon* was used as an outgroup. The tree was constructed from ITS sequence regions of *Loliinae* species and *B. distachyon* with PhyML implemented in SeaView [5]. Detailed phylogeny of subgenus Schedonorus is depicted and shows the relationships of fescue and ryegrass species in this lineage (highlighted in light yellow)

Grasses (Poaceae) are an important source of food for domestic animals worldwide and perform important ecological and environmental functions. The tribe Poeae is the largest tribe in family Poaceae, and species from its largest subtribe, Loliinae, grow in a range of habitats, including wetlands, dry areas, and regions with cold and temperate climates; some are even well adapted to the extreme conditions of mountain, arctic, and sub-Antarctic regions [1]. The subtribe Loliinae comprises a cosmopolitan genus *Festuca* and its satellite genera [2, 3]. *Festuca* is the largest genus of the family Poaceae, containing more than 600 species, and Torrecilla and Catalán [4] discriminated its two main evolutionary lines: broad leaved and fine leaved (Fig. 1). Broad-leaved *Festuca* species (hereafter “fescues”) include the subgenus Schedonorus, which gave rise to *Lolium* species (hereafter “ryegrasses”), a sister group of fescues (Fig. 1) [1]. The evolution of grasses, including *Loliinae*, has been accompanied by frequent polyploidization and hybridization events, and about 70% of grass species are polyploid [6]. The species of Loliinae have large genomes ranging from 2.6 Gbp/1C to 11.8 Gbp/1C [7, 8].

This study focuses on species from the subgenus Schedonorus, a complex of species with various ploidy levels [7, 9] that includes important species widely used for forage and turf. Although some Schedonorus species are diploid, such as *Festuca pratensis* Huds. (2n = 2x = 14) and *Lolium multiflorum* Lam. (2n = 2x = 14) and *L. perenne* L. (2n = 2x = 14), the majority of species are allopolyploid [10, 11], including tetraploids *F. glaucescens* Boiss. (2n = 4x = 28) and *F. mairei* St. Yves (2n = 4x = 28) and hexaploids *F. arundinacea* Schreb. (2n = 6x = 42) and *F. gigantea* (L.) Vill. (2n = 6x = 42) [3, 11]. Fescues are more tolerant than ryegrasses of abiotic stresses, provide high-quality forage for livestock, and are grown especially for turf purposes. In contrast, ryegrasses are characterized by high yield and excellent nutritional value and are mostly cultivated as pasture. Artificial intergeneric hybrids of fescue and ryegrass species have been developed to combine the most favorable characteristics of both genera [12–14].

Although fescues and ryegrasses have been intensively studied, their evolution and the origin of most polyploid representatives remain obscure [11, 15, 16]. Like in other species with large genomes, the nuclear genomes of fescues and ryegrasses include a large number and variety of repetitive DNA sequences [17, 18]. Their amplification in the genome, accompanied by interspecific hybridization and polyploidization, has expanded the genome size [19–24]. However, these processes have likely been counterbalanced by recombination-based mechanisms that have removed substantial parts of nuclear genomes [25–27].

Repetitive DNA elements may play different roles in a nuclear genome. Tandem organized ribosomal RNA genes and telomeric sequences are the key components of nucleolar organizing regions and chromosome termini, respectively. Centromeric regions in *Arabidopsis*, *Brachypodium*, rice, and maize are partly formed from specific satellite DNAs with ~ 130 bp long units [28–31], whereas in other plant species, including cereals, these regions are formed by large blocks of Ty3/gypsy retrotransposons containing chromodomain [29, 32–34]. In *F. pratensis*, a putative long terminal repeat (LTR) element localizing preferentially to centromeric regions has been identified [35]. In addition to elucidating the molecular organization of chromosome domains, characterization of repetitive parts of nuclear genomes helps in the development of cytogenetic markers [21, 35, 36]. Repetitive DNA sequences are also used extensively to study genetic diversity and processes of genome evolution and speciation [37–40].

The main goal of the present work was to elucidate the repetitive landscape and its impact on genome size and genome divergence in closely related land grasses, including natural polyploid species. We characterized repetitive DNA sequences in the nuclear genomes of 10 representatives of fescues and ryegrasses. We performed global analyses of repetitive DNA sequences and characterized their abundance and variability after partial Illumina sequencing. Moreover, we characterized and assembled the DNA sequence of an LTR element that was highly enriched in centromeric and (peri)centromeric chromosome regions in all 10 genotypes. Co-localization of the centromere-specific histone H3 variant CENH3 with the LTR element indicated its role in centromere function.

## Results

### Genome size estimation

**Fig. 2:**
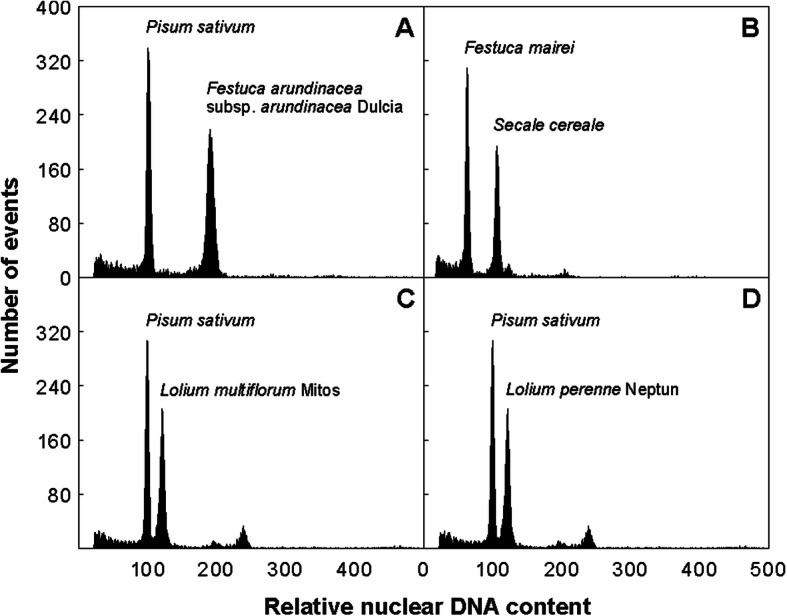
Estimation of nuclear genome size. Histograms of propidium iodide–stained nuclei corresponding to relative nuclear DNA content obtained after flow cytometric analyses of *F. arundinacea* subsp. *arundinacea* (**a**), *F. mairei* (**b**), *Lolium multiflorum* cv. Mitos (**c**), and *L. perenne* cv. Neptun (**d**). *Pisum sativum* cv. Ctirad (2C = 9.09 pg) and *Secale cereale* cv. Dankovske (2C = 16.19 pg), respectively, were used as internal reference standards. The ratio of relative G1 peak positions was used to calculate DNA amounts of the fescue and ryegrass accessions

Flow cytometric analysis of propidium iodide–stained nuclei was conducted to estimate nuclear DNA content (Fig. 2). Because of the large differences in genome size between the species analyzed, two internal reference standards were used: *Pisum sativum* cv. Ctirad (2C = 9.09 pg DNA) [41] and *Secale cereale* cv. Dankovske (2C = 16.19 pg DNA) [41]. All histograms of relative DNA content represented two dominant peaks corresponding to G1 nuclei of the sample and the standard. The 2C nuclear DNA content thus determined ranged from 5.32 pg in *L. multiflorum* to 20.17 pg in *F. gigantea*. The monoploid genome (1Cx) ranged in size from 2.43 in *F. mairei* to 3.36 pg in *F. gigantea* (Table 1). The remaining representatives of fescues and ryegrasses had similar 1Cx sizes (~ 2.7 Gb).

**Table 1:** Flow cytometric estimation of nuclear genome size

Species	Accession name	Code	Ploidy level	2C nuclear DNA content		Monoploid genome size (1Cx)	
				Mean [pg]	± SD	[pg]	[Mbp]
*Festuca pratensis*	Fure	FPF	2n = 2x = 14	6.4	0.04	3.2	3130
*Festuca pratensis*	Westa	FPW	2n = 4x = 28	12.79	0.09	3.2	3127
*Festuca arundinacea ssp. arundinacea*	Dulcia	FAR	2n = 6x = 42	16.85	0.24	2.81	2747
*Festuca arundinacea ssp. glaucescens*	–	FGL	2n = 4x = 28	10.79	0.07	2.7	2638
*Festuca gigantea*	GR 11759	FGI	2n = 6x = 42	20.17	0.14	3.36	3288
*Festuca mairei*	GR 610941	FMA	2n = 4x = 28	9.73	0.05	2.43	2379
*Lolium multiflorum*	Lm2	LM2	2n = 2x = 14	5.32	0.03	2.66	2601
*Lolium multiflorum*	Mitos	LMM	2n = 4x = 28	11.13	0.05	2.78	2721
*Lolium perenne*	GR 3320	LP2	2n = 2x = 14	5.54	0.03	2.77	2709
*Lolium perenne*	Neptun	LPN	2n = 4x = 28	10.94	0.15	2.74	2675

### Repeat composition and comparative analyses of repetitive DNA sequences

Interspecific comparisons, reconstruction, and quantification of major repeat families were performed with the RepeatExplorer pipeline [42]. The process, which involved grouping orthologous repeat families from all analyzed species in the same cluster, facilitated the assembly, identification, and quantification of individual repeat elements.

**Fig. 3:**
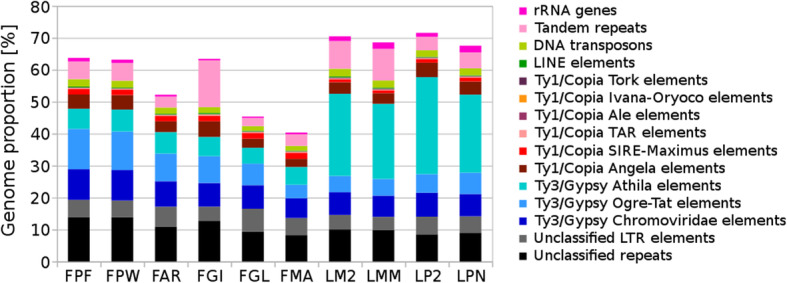
Genome proportion of the most abundant DNA repeats. The genome proportion of individual repeat types was obtained as a ratio of reads specific to individual repeat types to all reads used for clustering analyses by the RepeatExplorer pipeline. Diploid *Festuca pratensis* cv. Fure (FPF); tetraploid *F. pratensis* cv. Westa (FPW); hexaploid *F. arundinacea* subsp. *arundinacea* (FAR); hexaploid *F. gigantea* (FGI); tetraploid *F. glaucescens* (FGL); tetraploid *F. mairei* (FMA); diploid cv. Kuri1 of *L. multiflorum* (LM2); tetraploid cv. Mitos of *L. multiflorum* (LMM); diploid *L. perenne* (LP2); tetraploid cv. Neptun of *L. perenne* (LPN)

In all accessions, LTR retroelements were the most abundant component of the nuclear genome (Table 2, Fig. 3). Ty3/gypsy elements were more than 4 times more abundant than Ty1/copia retrotransposons (Table 2). The biggest difference in copy number between fescues and ryegrasses was for an LTR element from the Athila clade. Whereas the nuclear genomes of both *Lolium* species were enriched for the element, which accounted for ~ 25–30% of their genomes, the orthologous Athila element accounted for only ~ 5–7% of the nuclear genomes of fescues (Table 2). A relatively large part of the genomes was represented by unclassified LTR sequences, which indicates a high frequency of unique LTR sequences. DNA transposons and long interspersed nuclear element (LINE) elements were found in low copy numbers, and tandem repeats accounted for 1.5% to more than 8% of the genome sequences (Table 2, Fig. 3).

**Table 2:** Proportion of repetitive DNA sequences identified de novo

Repeat		Lineage/class	Proportion of repeat in monoploid genomes [%]									
			FPF	FPW	FAR	FGI	FGL	FMA	LM2	LMM	LP2	LPN
**LTR retroelements**	***Ty1/Copia***	Maximus-SIRE	1.72	1.65	1.69	1.78	1.84	1.93	0.89	0.87	1.16	1.25
		Angela	4.43	4.53	3.33	4.86	2.83	2.54	3.63	3.32	4.52	4.13
		TAR (Tont)	0.3	0.27	0.28	0.30	0.31	0.34	0.28	0.25	0.24	0.25
		Tork (Tnt)	0.05	0.04	0.05	0.05	0.05	0.06	0.07	0.07	0.08	0.07
		Ale (Hopscotch)	0.1	0.07	0.07	0.07	0.04	0.03	0.22	0.22	0.14	0.14
		Ivana-Oryoco	0.05	0.05	0.03	0.07	0.02	0.02	0.03	0.02	0.01	0.02
		**Total*Ty1/Copia***	**6.65**	**6.61**	**5.45**	**7.13**	**5.09**	**4.92**	**5.12**	**4.75**	**6.15**	**5.86**
	***Ty3/Gypsy***	Athila	6.32	6.88	6.73	6.02	4.96	5.56	25.69	23.54	30.33	24.4
		Chromovirideae	9.6	9.57	7.97	7.40	7.35	6.17	7.11	6.63	7.49	6.97
		Ogre-Tat	12.61	12.03	8.65	8.40	6.76	4.22	5.10	5.20	5.83	6.68
		**Total*Ty3/Gypsy***	**28.53**	**28.48**	**23.35**	**21.82**	**19.07**	**15.95**	**37.90**	**35.37**	**43.65**	**38.05**
**Unclassified LTR elements**			**5.51**	**5.15**	**6.35**	**4.43**	**7.14**	**5.35**	**4.55**	**4.14**	**5.54**	**5.15**
**Other**	**LINE**		**0.26**	**0.27**	**0.29**	**0.37**	**0.27**	**0.23**	**0.34**	**0.31**	**0.20**	**0.23**
	**DNA transposons**		**2.35**	**2.16**	**1.95**	**1.81**	**1.44**	**1.45**	**2.38**	**2.25**	**2.08**	**2.15**
	**Tandem repeats**		**5.52**	**5.53**	**3.41**	**14.63**	**2.55**	**3.63**	**8.67**	**9.86**	**4.20**	**4.99**
	**rRNA genes**		**1.13**	**1.07**	**0.57**	**0.50**	**0.43**	**0.56**	**1.48**	**2.03**	**1.23**	**2.10**
**Unclassified repeats**			**13.79**	**13.94**	**10.82**	**12.76**	**9.39**	**8.29**	**10.04**	**9.86**	**8.51**	**9.02**

**Fig. 4:**
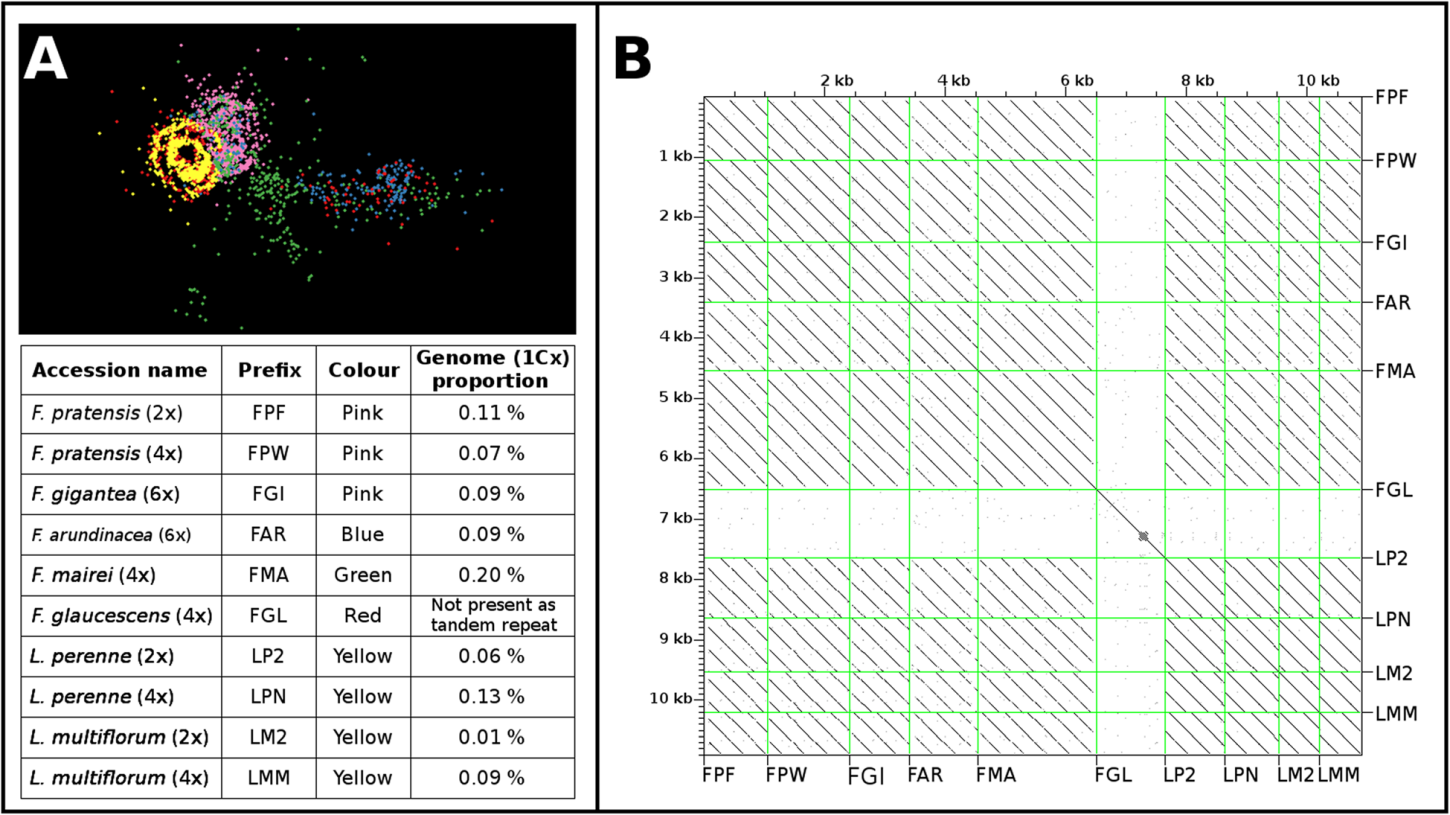
Tandem organized repeat sequences identified in cluster CL102. **a** Graphical layout of cluster CL102. **b** Dot-plot analyses show the presence of homologous tandem organized units (parallel lines) of DNA repeats identified in cluster CL102 in all species except *F. glaucescens*, in which the assembled sequence contigs did not represent tandem organized sequences

**Fig. 5:**
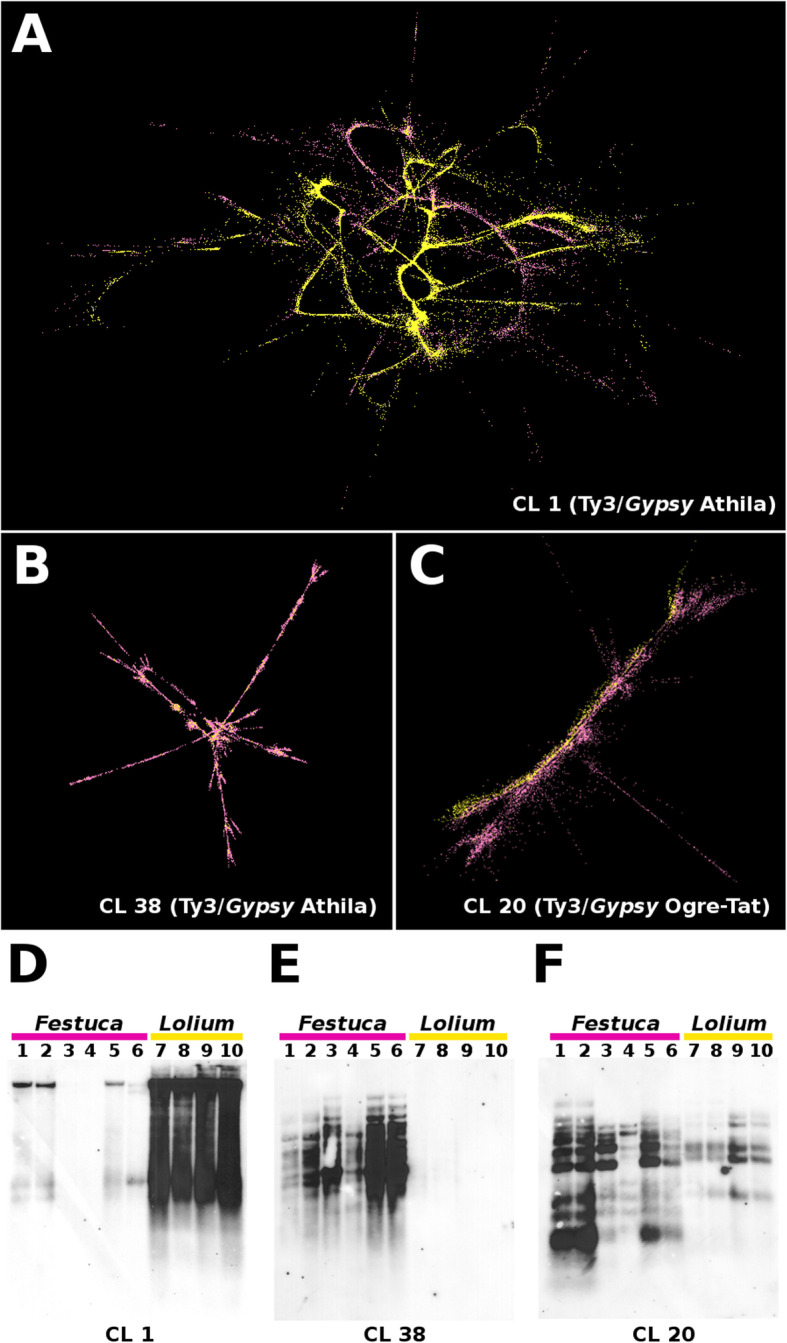
Graphical layouts of selected DNA repeats and their validation by Southern blotting. Graphical layouts were obtained after clustering analyses done by RepeatExplorer. **a** Cluster CL1. **b** Cluster CL38 containing the Ty3/gypsy Athila element. **c** Cluster CL20 containing Ty3/gypsy Ogre/Tat elements. Sequencing reads from *Festuca* species are in pink, whereas sequencing reads from *Lolium* species are in yellow. **d**–**f** Validation of clustering results by Southern hybridization with sequences derived from clusters CL1, CL38, and CL20. Lanes contained genomic DNA digested by *Hae*III restriction endonuclease. Lane 1: *F. pratensis* cv. Fure (2n = 2x = 14); lane 2: *F. pratensis* cv. Westa (2n = 4x = 28); lane 3: *F. arundinacea* subsp. *arundinacea* (2n = 6x = 42); lane 4: *F. gigantea* (2n = 6x = 42); lane 5: *F. glaucescens* (2n = 4x = 28); lane 6: *F. mairei* (2n = 4x = 28); lane 7: *L. multiflorum* cv. Mitos (2n = 4x = 28); lane 8: *L. multiflorum* cv. Kuri1 (2n = 2x = 14); lane 9: *L. perenne* cv. Neptun (2n = 4x = 28); lane 10: *L. perenne* GR 3320 (2n = 2x = 14)

Comparative analyses with RepeatExplorer showed that most clusters of orthologous repeat families contained reads from all accessions and that a large number of similar sequences were present in fescues and ryegrasses. Among the fescues, *F. mairei* and *F. glaucescens* showed the lowest similarity in DNA repeats. The composition as well as the abundance of DNA repeats in ryegrasses were highly conserved. Tandem organized repeats were the most diverged elements among the fescues and ryegrasses studied, and some of the repeats were species specific (Fig. 4, Additional file 1: Table S1). In addition to tandem repeats, some small sequence clusters contained reads from only a few species. Species-specific variants of the majority of repetitive elements within and between fescues and ryegrasses were identified only after detailed analyses of individual repeat clusters with SeqGrapheR (Fig. 5a–c). Detailed analyses revealed the presence of species-specific DNA contigs, which may be used to develop molecular and cytogenetic markers.

To confirm the differences determined in silico, we analyzed selected repetitive DNA elements using Southern hybridization. We designed specific probes for those DNA repeats that seemed to have species-specific variants. A probe for the Ty3/gypsy Athila element that was reconstructed in cluster CL1 and showed the largest copy number variation between fescues and ryegrasses (Table 2) gave strong hybridization signals on genomic DNA from ryegrasses but no or weak signals on DNA from fescues (Fig. 5d). Similarly, a probe for the Ty3/gypsy Athila element that was reconstructed in cluster CL38 and contained mostly *Festuca* sequence reads (Fig. 5b) provided strong visible signals only with fescue genomic DNA (Fig. 5e). Finally, Southern hybridization was performed with a probe for the Ty3/gypsy Ogre-Tat retrotransposon, identified in cluster CL20. The probe, which was designed from contigs representing fescues (Fig. 5c), provided strong hybridization signals on all fescues analyzed and low intensity signals on ryegrasses (Fig. 5f). In general, the signal intensities obtained after Southern hybridization corresponded to the copy numbers identified in silico.

### Centromere composition

**Fig. 6:**
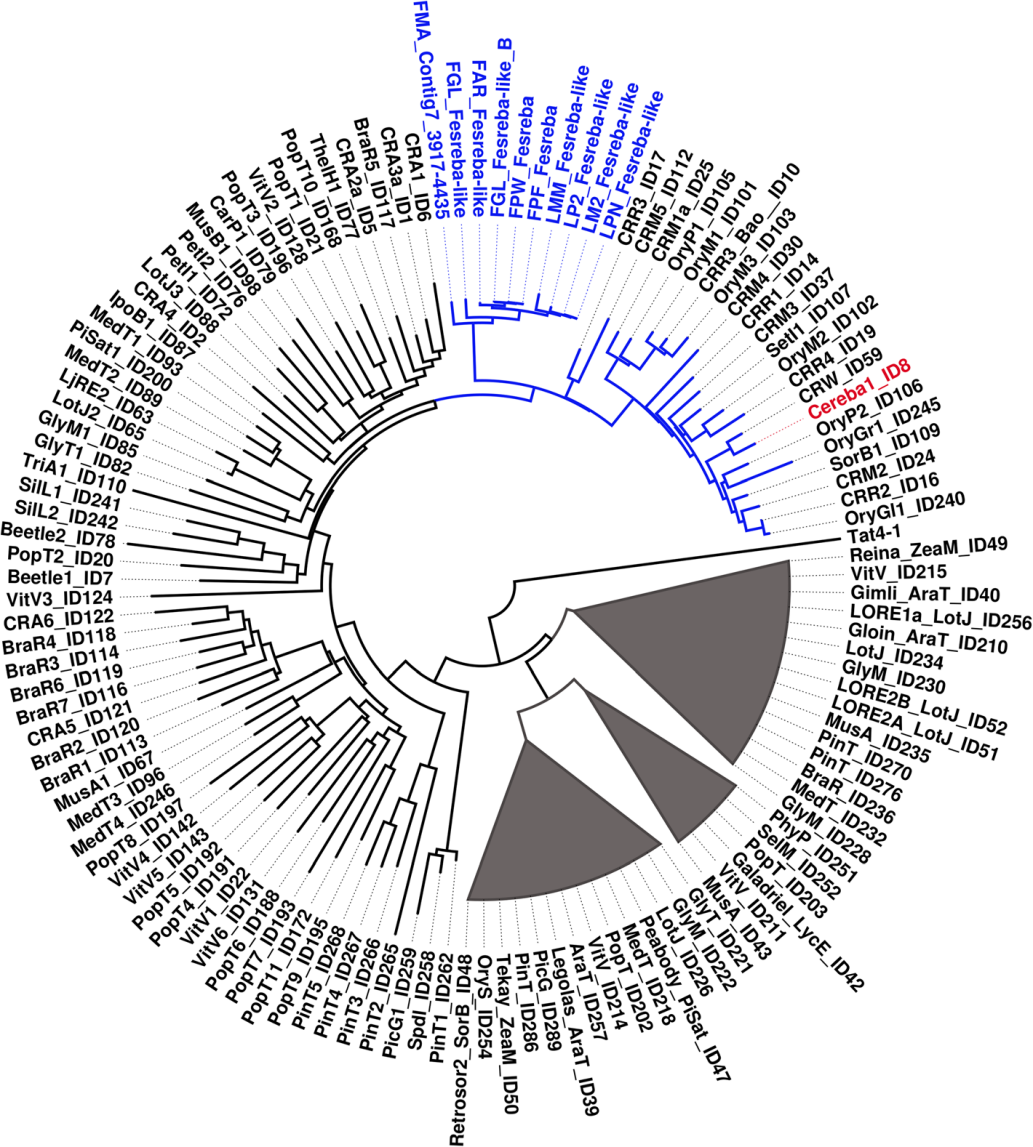
Phylogenetic tree of Chromoviridae elements. The tree was constructed from a Jukes-Cantor distance matrix of the reverse transcriptase domains of Ty3/gypsy Chromoviridae elements described in Neumann et al. [34] and Fesreba elements identified in the present work with BioNJ implemented in Seaview [5]. The tree was rooted on the Ty3/gypsy/Tat element. The subclade of the Cereba element (in red) and other closely related elements identified in different plant species are labeled in blue. Fesreba elements identified in fescues and ryegrasses are also marked in blue

Partial genome sequence data obtained using Illumina sequencing technology made it possible to reconstruct nearly complete centromeric LTR elements in all 10 accessions of fescues and ryegrasses. Detailed characterization of the element called Fesreba confirmed that it belongs to the Ty3/gypsy Chromoviridae lineage. Phylogenetic analyses of its reverse transcriptase (RT) domain showed a close relationship with the Cereba element (Fig. 6), which was identified earlier in barley (*Hordeum vulgare*) [43].

Southern hybridization with a probe for the RT domain of Fesreba and a probe for its LTR region [35] showed their presence in all fescues and ryegrasses included in this work (Additional file 2: Fig. S1). Similar hybridization patterns indicated sequence conservation between Fesreba repetitive DNA elements in these species. The results were supported by in silico data, which showed high similarity at the DNA sequence level (most abundant copies of Fesreba shared at least 92% similarity at the DNA level within and between fescues and ryegrasses) but lower abundance in ryegrasses. To confirm the differences in Fesreba copy number, we performed quantification for the RT domain and LTR sequence using droplet digital polymerase chain reaction (ddPCR). The results confirmed a two-fold higher copy number of Fesreba in fescues compared to ryegrasses (Additional file 3: Table S2). The assay also showed that the majority of genotypes analyzed contained 5 to 50 times more copies of the LTR region of Fesreba than its coding region (Additional file 3: Table S2).

**Fig. 7:**
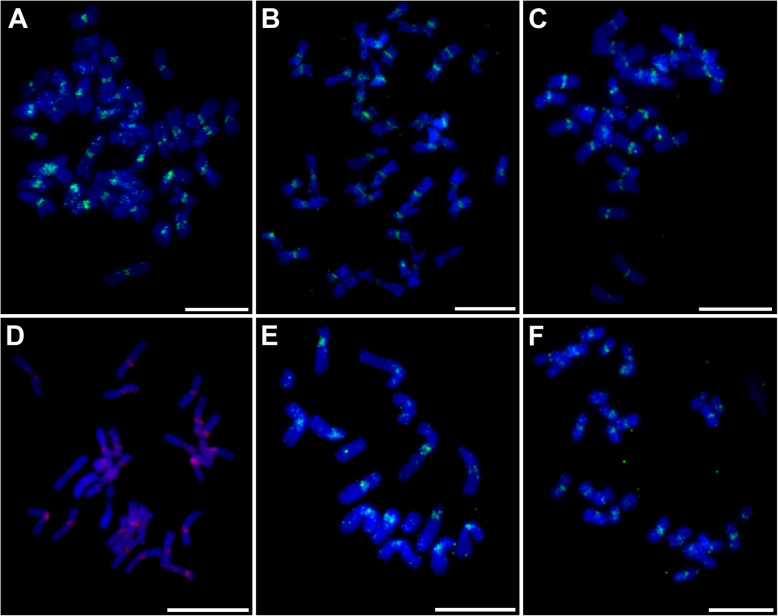
Localization of the centromeric LTR retrotransposon Fesreba on mitotic metaphase chromosomes. Localization was performed in *Festuca* and *Lolium* species with fluorescence in situ hybridization (yellow-green or violet signals) with a probe for the reverse transcriptase domain of the Fesreba element. **a***F. arundinacea* subsp*. arundinacea* (2n = 6x = 42). **b***F. gigantea* (2n = 6x = 42). **c***F. mairei* (2n = 4x = 28). **d***F. pratensis* cv. Westa (2n = 4x = 28). (E) *L. perenne* GR 3320 (2n = 2x = 14). **f***L. multiflorum* cv. Mitos (2n = 4x = 28). Chromosomes were counterstained with DAPI (blue). The bar corresponds to 10 μm

To confirm preferential localization of Fesreba to centromeric chromosome regions, we conducted fluorescence in situ hybridization (FISH) on mitotic metaphase plates with probes derived from its RT domain and LTR region. In all fescues and ryegrasses, both probes localized preferentially to centromeric regions of all chromosomes (Fig. 7). Whereas the hybridization signals of the RT domain were observed almost exclusively in centromeric regions, a probe derived from the non-coding LTR region resulted in stronger signals in centromeric and/or pericentromeric regions and weak signals along the chromosomal arms, as shown previously in *F. pratensis* [35]. Weak signals of the LTR part of Fesreba in distal parts of chromosomes indicate the presence of unique LTRs spread over the genome and correspond to a higher copy number of the LTR non-coding part of Fesreba compared to its coding sequence.

**Fig. 8:**
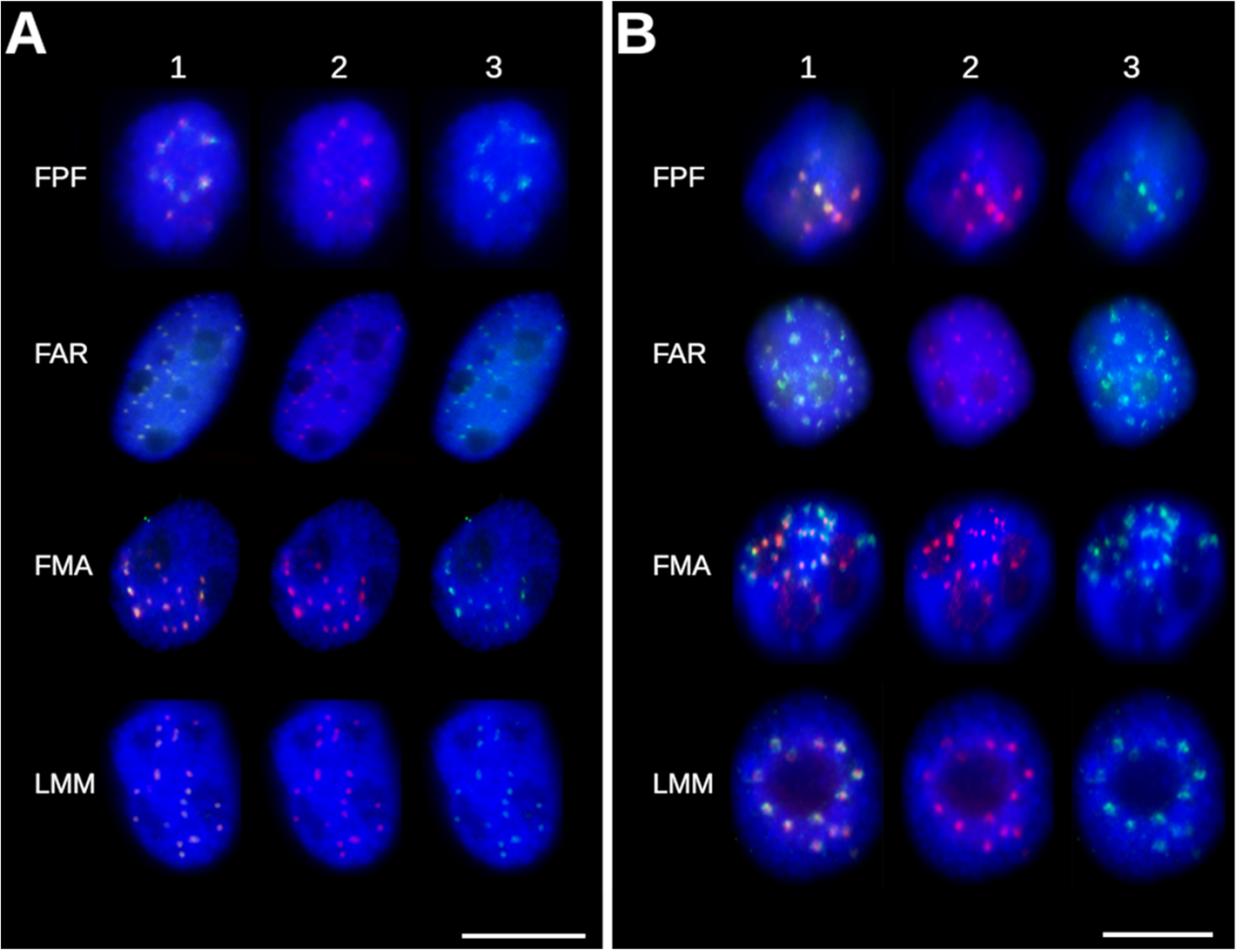
Co-localization of CENH3 with the Fesreba element in *Festuca* and *Lolium*. Combination of immunolocalization of the histone H3 variant CENH3 (red) and FISH on interphase nuclei with probes for: **a** the reverse transcriptase (RT) domain (green); and **b** the non-coding LTR part of the Fesreba element (green). *F. pratensis* cv. Fure (FPF); *F. arundinacea* subsp*. arundinacea* (FAR); *F. mairei* (FMA); *L. multiflorum* cv. Mitos (LMM). Column 1 shows merged images, column 2 shows CENH3 signals (red), and column 3 shows FISH signals corresponding to the Fesreba element. In all accessions, the signals of CENH3 and FISH probes are overlapping. Nuclei were counterstained with DAPI (blue). The bar corresponds to 10 μm

In addition to the fescues and ryegrasses included in this study, FISH was performed with the same probes on mitotic metaphase plates from related grass species, oat, barley, rye, bread wheat, and *Aegilops tauschii*. High homology of the RT coding domain resulted in successful in situ localization in all species. However, the probe specific to the LTR region of Fesreba provided visible signals only in *A. sativa* (Additional file 4: Fig. S2). Finally, immunostaining with the centromere-specific histone H3 variant CENH3 [44] in combination with FISH with probes for the RT domain and LTR region of Fesreba resulted in overlapping signals in all fescues and ryegrasses studied (Fig. 8, Additional file 5: Fig. S3).

## Discussion

Because of genome shock, the 1Cx size of polyploid species is often, but not always, lower than that of their progenitors [25, 45]. In this study, we performed comparative analyses of repeatomes and analyzed the impact of DNA repeats on genome size in a set of *Festuca* and *Lolium* species differing in ploidy. The set comprised hexaploids *F. arundinacea* subsp. *arundinacea* and *F. gigantea*; tetraploids *F. glaucescens* and *F. mairei*; and artificial autotetraploids *F. pratensis* cv. Westa, *L. multiflorum* cv. Mitos, and *L. perenne* cv. Neptun developed in breeding programs. We estimated nuclear DNA amounts using flow cytometry, and a test of normality confirmed that the data set had a normal distribution. Our results suggest possible genome changes in hexaploid *F. arundinacea* and tetraploid ryegrasses compared to their probable progenitors. Although the differences in the 1Cx size of natural polyploid *F. arundinacea* and its probable parents (*F. pratensis* and *F. glaucescens*) are small, they are statistically significant (*P* < 0.01). The same is true for tetraploid ryegrass cultivars obtained after polyploidization. Genome downsizing was detected in the case of *F. arundinacea* (~ 2% difference between expected and estimated values) and tetraploid *L. perenne* (~ 1% decrease). In the tetraploid cultivar of *L. multiflorum*, a slight increase in genome size (~ 4%) was detected, corresponding with Kopecký et al. [8]. In the case of tetraploid fescue cultivars obtained after polyploidization, no statistically significant difference in 1Cx value was found (*P* > 0.01).

DNA retrotransposons are major contributors to the variation in nuclear genomes in plants [24, 46, 47]. Various approaches and tools have been developed to study these important parts of nuclear genomes, one of them being RepeatExplorer, which facilitates de novo repeat identification and characterization [42, 48]. The pipeline uses graph-based clustering and analyzes next-generation sequencing data to reconstruct and characterize DNA repeats in a particular species or to compare DNA repeat composition in different genotypes [23, 24, 49–51]. The pipeline has been frequently used to reconstruct DNA repeats in diversity studies, to create repeat databases for repeat masking [19, 46, 48], and to identify tandem organized repeats suitable as probes for molecular cytogenetics [35, 51–53].

Our work revealed that Ty3/gypsy elements had the highest impact on genome size in fescues and ryegrasses. Ty3/gypsy elements are also abundant in other *Poaceae* species, including wheat, rice, maize, and barley [8, 54–56]. In barley, about 50% of the genome is made up of 15 high-copy transposable element (TE) families, with elements of the Angela lineage (Ty1/copia family) being the most abundant and representing almost 14% of the genome [56]. The Ty3/gypsy superfamily is 1.5-fold more abundant than the Ty1/copia superfamily [56].

*Festuca* and *Lolium* genera comprise closely related complexes of species, and thus a high homology of DNA repeats was observed in this study. The main difference was the copy number. In *Lolium* species the Ty3/gypsy Athila LTR retroelement accounted for ~ 25% of the nuclear genomes, whereas in fescues it accounted for ~ 0.7% in tetraploids *F. glaucescens* and *F. mairei* and for ~ 6% in other fescues analyzed. This indicates a burst of Athila LTR element linked with *Lolium* speciation. Activation and integration of TEs (e.g., as a result of environment change) may lead to a rapid burst of the Athila element in a species-specific manner [46, 47, 57] and impact evolution and speciation [46, 58]. In some species, a rapid increase in the number of lineage-specific retroelements can also result in significant genome upsizing [24, 58–60], which was not observed in the fescues and ryegrasses included in our study.

Species-specific DNA elements identified in this work were represented by tandem organized repeats (Additional file 1: Table S1). Unique tandem repeats are also found in other plant species, and thanks to their genus or species specificity they have been widely used in molecular cytogenetics (e.g., to identify chromosomes using FISH) [61–64]. Tandem repeats originally identified in *F. pratensis* chromosome 4F are useful as probes for FISH to identify individual chromosomes of the species [18, 35] and in comparative karyotype analyses of its cultivars. The present work resulted in the identification of other putative tandem organized repeats, either genus or species specific (Additional file 1: Table S1). These observations expand the number of potential cytogenetic markers for comparative karyotyping and identification of chromosomes in other fescue and ryegrass species.

Although the sequencing of *F. pratensis* chromosome 4F revealed a relatively high number of tandem repeats, none of them localized to chromosome centromeric regions [18, 35]. However, the mapping of other types of DNA repeats on mitotic metaphase chromosomes showed preferential localization of one uncharacterized DNA element CL38 to centromeric regions of *F. pratensis* chromosomes [35]. In this work, the entire DNA element homologous to the CL38 repeat was reconstructed and its nature was clarified. Phylogenetic analyses of its coding domains (Fig. 6) confirmed close relationships with other plant centromeric elements of Ty3/gypsy Chromoviridae lineage, such as Cereba-like elements [43]. Preferential localization of the Cereba element to centromeric regions of barley chromosomes was shown by Hudakova et al. [33], and more complex study of centromere-specific elements belonginging to the lineage of Centromeric retrotransposons in maize (CRM) of the Ty3/gypsy family in larger set of plant species followed [20, 34]. These studies imply a role for TEs at the structural level and their impact on centromere structure. Li et al. [65] showed that the Cereba element was strongly associated with the histone H3 variant CENH3, which plays a role in centromere function. Co-localization of the centromere-specific element Fesreba, reconstructed in this work with histone CENH3 (Fig. 8, Additional file 5: Fig. S3), indicates a role for this element in the function of fescue and ryegrass centromeres as well.

## Conclusions

Partial sequencing of genomes of 10 fescues and ryegrasses revealed various types of retrotransposons as the most abundant repeat. These comparative repeatome analyses increase knowledge of genome organization in fescues and ryegrasses and confirm close relationships between *Festuca* and *Lolium*. The most striking difference was observed for the Athila element, which was ~ 5 times more abundant in *Lolium* than *Festuca*. Highly diverged DNA repeats were represented by tandem organized repeats, which are candidates for species-specific cytogenetic markers. In addition to tandem repeats, other species-specific variants of the majority of repetitive DNA sequences within and between fescues and ryegrasses were identified. A nearly complete LTR element Fesreba was assembled and found to be highly enriched in centromeric and (peri)centromeric chromosome regions in all species. A combination of FISH with a probe for Fesreba and immunostaining with CENH3 antibody showed their co-localization and indicated a possible role of Fesreba in centromere function.

## Methods

### Plant material

*Lolium perenne* GR3320 (2n = 2x = 14), *Festuca arundinacea subsp. arundinacea* (2n = 6x = 42), *Festuca gigantea* GR11759 (2n = 6x = 42), and *Festuca mairei* GR610941 (2n = 4x = 28) were obtained as seeds from the Leibniz Institute of Plant Genetics and Crop Plant Research (Gatersleben, Germany) gene bank. Seeds of *Festuca pratensis* cv. Fure (2n = 2x = 14) were obtained from Dr. Arild Larson (Graminor, Norway). *Lolium perenne* cv. Neptun (2n = 4x = 28), *Lolium multiflorum* cv. Kuri1 (2n = 2x = 14), and two commercially available cultivars, *Lolium. multiflorum* cv. Mitos (2n = 4x = 28) and *Festuca pratensis* cv. Westa (2n = 4x = 28), were obtained from Dr. Vladimír Černoch (DLF Seeds, Czech Republic). *Festuca glaucescens* genotype C-45 (2n = 4x = 28) was obtained from Seed Bank, W. Reg. P. I. Station, Pullman, WA.

Seeds of barley (*Hordeum vulgare*) cv. Morex, rye (*Secale cereale*) cv. Dánkowskie Diament, and oat (*Avena sativa*) cv. Atego were obtained from the Leibniz Institute of Plant Genetics and Crop Plant Research gene bank. Seeds of *Triticum aestivum* cv. Chinese Spring were obtained from Professor Takashi R. Endo (Kyoto University, Japan), and seeds of *Aegilops tauschii* (lineage AL 8/78; collected by V. Jaaska, University of Estonia, Tartu, Estonia) were provided by Dr. Valárik (Institute of Experimental Botany, Czech Republic). Seeds of pea (*Pisum sativum* cv. Ctirad) and rye (*Secale cereale* cv. Dankovske), which served as internal reference standards in flow cytometric analyses, were provided by one of us (JD) and are available at the Institute of Experimental Botany, Czech Republic (https://olomouc.ueb.cas.cz/en/technology/flow-cytometry-1/reference-dna-standards).

### Estimation of nuclear genome size

Nuclear DNA amounts were determined according to Doležel et al. [66] following the two-step procedure of Otto [67] with modifications. Samples of isolated nuclei stained with propidium iodide were analyzed with a Sysmex CyFlow Space flow cytometer (Sysmex Partec, Münster, Germany) equipped with a 532 nm laser. Two reference standards were used to estimate DNA amounts in absolute units. Pea (*Pisum sativum* cv. Ctirad; 2C = 9.09 pg DNA) [41] served as an internal standard for estimating DNA content in all accessions except *F. mairei*, for which rye (*Secale cereale* cv. Dankovske; 2C = 16.19 pg DNA) [41] was used. Three plants were measured per accession, and each plant was analyzed three times on three different days. At least 5000 nuclei per sample were analyzed. Nuclear amounts were calculated from measurements of individual samples as follows: 2C nuclear DNA content (pg) = 2C nuclear DNA content of reference standard × sample G_1_ peak mean / standard G_1_ peak mean.

Mean nuclear DNA content (2C) was estimated for each plant, with 1 pg DNA equal to 0.978 × 10^9^ bp [68]. The statistical significance of the differences between 1Cx sizes was determined with one-way ANOVA. Analyses were conducted with NCSS 97 (Statistical Solutions, Cork, Ireland). The significance level α = 0.01 was used.

### Phylogenetic analyses

Phylogenetic analyses of Loliinae subtribe were based on data published by Catalán et al. [3]. Sequences of ITS regions were downloaded from the NCBI GenBank (GB codes: AF303401–407, AF303410–416, AF303418–419, AF303421–425, AF303428, AF478475–476, AF478478–491, AF478493, AF478498–499, AF519975–981, AF519983, AF532937, AF532939–948, AF532951–952, AF532954, AF532956–960, AF532962–963, AF543514, AF548028, AJ240143, AJ240146, AJ240148, AJ240153, AJ240155–157, AJ240160, AJ240162, AY099007, AY118087–088, AY118090–092, AY118094–096, AY228161). *Brachypodium distachyon* (GB code AF303339) was used as an outgroup species. Multiple sequence alignment was done with MAFFT v7.029 (−-localpair --maxiterate 1000) [69], and phylograms were constructed with PhyML 3.0 [70] implemented in SeaView v5.0.2 [5]. Approximate likelihood ratio tests [71] were performed to assess branch support. Final phylogenetic trees were depicted with FigTree (http://tree.bio.ed.ac.uk/software/figtree/).

### Illumina sequencing and data analyses

Genomic DNA was isolated with the NucleoSpin PlantII kit (Macherey-Nagel, Düren, Germany) according to the manufacturer’s recommendations and used to prepare Illumina libraries with a Nextera® DNA Sample Preparation Kit (Illumina, San Diego, CA, USA). Briefly, 50 ng DNA was fragmented, purified, and amplified according to the protocol. The DNA concentration in individual libraries was measured with a Qubit fluorometer, adjusted to an equal molar concentration, and pooled prior to sequencing. DNA sequencing was done with an Illumina MiSeq with either single or paired-end sequencing to produce up to 500 bp reads. Sequence reads were deposited in the Sequence Read Archive (BioProject ID: PRJNA601325, accessions SAMN13866227, SAMN13866228, SAMN13866229, SAMN13866230, SAMN13866231, SAMN13866232, SAMN13866233, SAMN13866234, SAMN13866235, SAMN13866236).

Illumina reads were trimmed for adapters and for quality with the FASTX-Toolkit (−q 20 -p 90; http://hannonlab.cshl.edu/fastx_toolkit/index.html). Detailed characterization of repeat families was performed with a stand-alone version of the RepeatExplorer pipeline [37] running on an IBM server with 16 processors, 100 Gb RAM, and 17 Tb disk space. In the first step, comparative analyses of repetitive parts of the genomes were performed with the RepeatExplorer pipeline according to Novák et al. [49]. Random data sets represented the same amount of reads (0.5× coverage of individual accessions) were used to reconstruct repetitive elements using a graph-based method according Novák et al. [48]. The RepeatExplorer pipeline led to the characterization of assembled sequences using different tools (e.g., BLASTN and BLASTX, phylogenetic analysis) [37, 48]. Tandem organized repeats were identified with Dotter [72].

In the second step, the RepeatExplorer pipeline was applied to a merged data set containing all species marked by specific prefixes to perform comparative analyses [49]. The results of the clustering were then used to create repetitive databases. Databases of Illumina reads were deposited in the Sequence Read Archive (accessions: SRX7566047–SRX7566056). Assembled contigs from different types of repetitive DNA elements are publicly available online (https://olomouc.ueb.cas.cz/en/content/dna-repeats) and in the Dryad digital repository (doi:10.5061/dryad.xksn02vch).

### Southern hybridization

Genomic DNA corresponding to 3 × 10^6^ copies of a 1Cx nuclear genome was digested by *Hae*III enzyme (New England Biolabs, Ipswich, MA, USA). DNA fragments were size-fractionated by electrophoresis in 1.2% agarose gel and then transferred onto Hybond™ N+ nylon membranes (GE Healthcare, Chicago, IL, USA). Probes were prepared with *F. pratensis* genomic DNA as a template and polymerase chain reaction (PCR) with biotin-labeled dUTP (Roche, Mannheim, Germany) and specific primers (Additional file 6: Table S3, Additional file 7: Fig. S4). Southern hybridization was performed at 68 °C overnight, and hybridization signals were detected with a Chemiluminescent Nucleic Acid Module (Thermo Fisher Scientific, Waltham, MA, USA) according to the manufacturer’s recommendations with 90% stringency. Hybridization signals were visualized with chemiluminiscent substrate on Medical X-Ray Film Blue (Agfa Healthcare, Mortsel, Belgium).

### ddPCR

Based on the assembled DNA contigs from the Fesreba retrotransposon, two restriction endonucleases with unique restriction sites in the retrotransposon (*Hpa*I and *Hpa*II) were identified and used for further analyses. Briefly, 3 μg genomic DNA was digested according to the manufacturer’s recommendations (Bio-Rad Laboratories, Hercules, CA, USA) and then diluted 1000-fold to reach a starter concentration of 0.06 ng/μl. ddPCR was performed on a QX200 Droplet Digital PCR machine (Bio-Rad Laboratories) following the manufacturer’s recommendations with EvaGreen Supermix (Bio-Rad Laboratories), template DNA, and specific primers for Fesreba (Additional file 6: Table S3). Three independent replicates were performed for every accession analyzed.

### Cytogenetic mapping and immunostaining

Cytogenetic mapping of selected repeats was done by FISH on mitotic metaphase plates. Chromosome spreads were prepared according to Křivánková et al. [35], and immunostaining was performed according to Neumann et al. [73]. Root tips were collected in ice water for 28 h; washed in LB01 buffer [74]; fixed in 3.7% formaldehyde for 25 min; and digested using 2% cellulose, 2% pectinase, and 2% cytohelicase in 1× phosphate-buffered saline (PBS) for 90 min at 37 °C. After the coverslip was removed, the preparations were washed in 1× PBS and then in PBS-Triton buffer (1× PBS, 0.5% Triton X-100, pH 7.4) for 25 min and then again in 1× PBS. For incubation with anti-grass CENH3 primary antibody [75], the slides were washed in PBS-Tween buffer (1× PBS, 0.1% Tween 20, pH 7.4) for 25 min and then incubated with anti-grass CENH3 primary antibody (diluted 1:200 in PBS-Tween) overnight at 4 °C. The next day the slides were washed in 1× PBS, CENH3 antibody was detected using the anti-Rabbit Alexa Fluor 546 secondary antibody (Thermo Fisher Scientific/Invitrogen) diluted 1:250 in PBS-Tween buffer for 1 h at room temperature, and washed 1× PBS. Before FISH, immunofluorescent signals were stabilized with ethanol:acetic acid (3:1) fixative and 3.7% formaldehyde for 10 min at room temperature. FISH was performed after three washes in 1× PBS.

Probes for FISH, derived from the RT and LTR regions of the Fesreba element, were labeled with digoxigenin-11-dUTP or biotin-16-dUTP (Roche Applied Science) using PCR with specific primers (Additional file 6: Table S3). FISH and detection of hybridization sites were performed according to Křivánková et al. [35]. The chromosomes were counterstained with 4′,6-diamidino-2-phenylindole (DAPI) and mounted in Vectashield (Vector Laboratories). The slides were examined with an Axio Imager.Z2 microscope (Carl Zeiss, Oberkochen, Germany) equipped with a Cool Cube 1 (Metasystems, Altlussheim, Germany) camera, and images were prepared with ISIS 5.4.7 (Metasystems). Final adjustments were made to figures in Adobe Photoshop 12.0.

## Supplementary information

**Additional file 1: Table S1.** List of clusters containing putative tandem repeats identified in *Festuca* and *Lolium*.

**Additional file 2: Fig. S1.** Southern blots for the RT domain and non-coding LTR part of the Fesreba element. Southern blots were made with probes for the reverse transcriptase domain (A) and non-coding LTR region (B) of the Fesreba element. Lanes contained genomic DNA digested by *Hae*III restriction endonuclease. Lane 1: diploid *F. pratensis* cv. Fure; lane 2: tetraploid *F. pratensis* cv. Westa; lane 3: hexaploid *F. arundinacea* subsp*. arundinacea*; lane 4: hexaploid *F. gigantea*; lane 5: tetraploid *F. glaucescens*; lane 6: tetraploid *F. mairei*; lane 7: tetraploid *L. multiflorum* cv. Mitos; lane 8: diploid *L. multiflorum* cv. Kuri1; lane 9: tetraploid *L. perenne* cv. Neptun; lane 10: diploid *L. perenne*.

**Additional file 3: Table S2.** Representation of the RT domain and non-coding part of the LTR region of the Fesreba element estimated by ddPCR. Copy numbers of the reverse transcriptase (RT) domain and non-coding part of the LTR region of the Fesreba element were estimated with droplet digital PCR. Values are averages of three independent experiments with standard deviations.

**Additional file 4: Fig. S2.** Localization of the centromeric LTR retrotransposon Fesreba on mitotic chromosomes with fluorescence in situ hybridization. Mitotic metaphase plates were hybridized with a probe for the reverse transcriptase domain of the Fesreba element (A, C, E, G, I) and with a combination of probes for the non-coding LTR part of the Fesreba element and 45S rDNA, which served as control (B, D, F, H, J). (A, B) *Avena sativa* cv. Atego (2n = 2x = 14). (C, D) *Secale cereale* cv. Dánkowskie Diament (2n = 2x = 14). (E, F) *Hordeum vulgare* cv. Morex (2n = 2x = 14). (G, H) *Triticum aestivum* cv. Chinese Spring (2n = 6x = 42). (I, J) *Aegilops tauschii* (2n = 2x = 14). Signals corresponding to 45S rDNA loci are marked by arrows. Hybridization signals of a probe for the LTR region of the Fesreba element were absent in all related species (D, F, H, J) except *A. sativa* (B). Chromosomes were counterstained with DAPI (blue). The bar corresponds to 10 μm.

**Additional file 5: Fig. S3.** Co-localization of CENH3 with the Fesreba element in three *Festuca* and three *Lolium* species. Immunolocalization of the histone H3 variant CENH3 (red) and FISH with probes for the reverse transcriptase (RT) domain and non-coding LTR part of the Fesreba element (green). *F. gigantea* (FGI); *F. glaucescens* (FGL); *F. pratensis* Westa (FPW); *L. multiflorum* Lm2 (LM2); *L. perenne* Neptun (LP2); *L. perenne* (LPN). Column 1 shows merged images, column 2 shows CENH3 signals (red), and column 3 shows FISH signals corresponding to the Fesreba element. In all accessions, the signals of CENH3 and FISH probes are overlapping. Nuclei were counterstained with DAPI (blue). The bar corresponds to 10 μm.

**Additional file 6: Table S3.** Primers used for PCR amplification of DNA repeats.

**Additional file 7: Fig. S4.** Original images of Southern hybridization depicted in Fig. 5 and Additional file 2: Fig. S1, respectively. Original images of Southern hybridization with sequences derived from cluster CL1 (A), cluster CL38 (B), and cluster CL20 (C) and with sequences for the reverse transcriptase domain (D) and non-coding LTR region (E) of the Fesreba element. Lanes contained genomic DNA digested by *Hae*III restriction endonuclease. Lane 1: diploid *F. pratensis* cv. Fure; lane 2: tetraploid *F. pratensis* cv. Westa; lane 3: hexaploid *F. arundinacea* subsp*. arundinacea*; lane 4: hexaploid *F. gigantea*; lane 5: tetraploid *F. glaucescens*; lane 6: tetraploid *F. mairei*; lane 7: tetraploid *L. multiflorum* cv. Mitos; lane 8: diploid *L. multiflorum* cv. Kuri1; lane 9: tetraploid *L. perenne* cv. Neptun; lane 10: diploid *L. perenne*.

## Data Availability

All relevant supporting data sets are included in the article and its additional files. The data sets supporting the conclusions in this article are available in the Sequence Read Archive (accessions: SRX7566047–SRX7566056) and Dryad repository (doi:10.5061/dryad.xksn02vch; https://datadryad.org/stash/share/8pm4qJ41tIJaXNd7EYkan125DKR-Vi8BINbF4HSobVg).

## Abbreviations

1C: Holoploid genome
1Cx: Monoploid genome
2C: Nuclear DNA amount in G1 nucleus prior to DNA replication
4F: Chromosome 4 of *Festuca pratensis* cv. Fure
bp: Base pairs
CENH3: Centromeric histone H3
CL: Cluster of orthologous sequences obtained by RepeatExplorer analysis
CRM: Centromeric retrotransposon in maize
Cy3: Cy3 fluorescent dye
DAPI: 4′,6-diamidino-2-phenylindole
ddPCR: Droplet digital polymerase chain reaction
DNA: Deoxyribonucleic acid
dUTP: 2′-deoxyuridine 5′-triphosphate
FAR: *Festuca arundinacea* Schreb. subsp. *arundinacea*
FGI: *Festuca gigantea* L. GR11759
FGL: *Festuca arundinacea* Schreb. subsp. *glaucescens*
FISH: Fluorescence in situ hybridization
FITC: Fluorescein isothiocyanate
FMA: *Festuca mairei* GR610941
FPF: *Festuca pratensis* Huds. cv. Fure
FPW: *Festuca pratensis* Huds. cv. Westa
G1: G1 phase of cell cycle
Gbp: Gigabase pairs
ID: Identity number
LINE: Long interspersed nuclear element
LM2: *Lolium multiflorum* cv. Kuri1
LMM: *Lolium multiflorum* Lam. cv. Mitos
LP2: *Lolium perenne* L. GR3320
LPN: *Lolium perenne* L. cv. Neptun
LTR: Long terminal repeat
μl: Microliter
NCBI: National Center for Biotechnology Information
ng: Nanogram
PBS: Phosphate-buffered saline
PCR: Polymerase chain reaction
pg: Picogram
pH: Potential of hydrogen
rDNA: Ribosomal DNA, DNA with ribosomal RNA genes
rRNA: Ribosomal RNA, RNA involved in structure of ribosomes and proteosynthesis
RT: Reverse transcriptase
SSC: Saline sodium citrate
TE: Transposable element
