# Supplementary material for: Comparative analyses of DNA repeats and identification of a novel Fesreba centromeric element in fescues and ryegrasses
Source: BMC Plant Biol. 2020 Jun 17;20:280. doi: 10.1186/s12870-020-02495-0 (PMC7302162; doi:10.1186/s12870-020-02495-0)
Supplement: Supplementary file 1 — Additional file 1: Table S1. List of clusters containing putative tandem repeats identified in Festuca and Lolium. [file 12870_2020_2495_MOESM1_ESM.docx]

**Table S1.** List of clusters containing putative tandem repeats identified in *Festuca* and *Lolium*.

| **Cluster^1^** | **Presence of tandem repeat in nuclear genome^2^** | | | | | | | | **Homology to sequences in GenBank (GenBank code)** |
| --- | --- | --- | --- | --- | --- | --- | --- | --- | --- |
|  | ***Festuca pratensis*** | ***Festuca gigantea*** | ***Festuca arundinacea*** | ***Festuca glaucescens*** | ***Festuca mairei*** | ***Lolium perenne*** | ***Lolium multiflorum*** |  | |
| CL 10 | *** | *** | ** | ** | ** | *** | *** | *F. pratensis* TR1 (JX624129) | |
| CL 60 | *** | *** | *** | *** | *** | *** | *** | *F. pratensis* TR4 (JX624130) | |
| CL 33 | ** | *** | ** | ** | ** | --- | --- | *F. pratensis* TR5 (JX624131) | |
| CL 87 | ** | *** | ** | ** | ** | ** | ** | *F. pratensis* TR6 (JX624132) | |
| CL 81 | ** | *** | ** | --- | --- | ** | **** | *F. pratensis* TR7 (JX624133) | |
| CL 102 | ** | ** | ** | *** | ** | ** | ** | *F. pratensis* TR12 (JX624135) | |
| CL 174 | *** | *** | *** | ** | ** | ** | ** | *F. pratensis* TR15 (JX624136) | |
| CL 37 | *** | *** | ** | ** | ** | ** | ** | 5S rDNA | |
| CL 35 | *** | *** | *** | *** | *** | *** | *** | --- | |
| CL 68 | ** | *** | ** | ** | ** | ** | **** | --- | |
| CL 71 | *** | *** | *** | ** | ** | *** | *** | --- | |
| CL 118 | ** | ** | ** | ** | ** | *** | *** | --- | |
| CL 147 | ** | *** | *** | *** | *** | --- | --- | --- | |
| CL 165 | --- | --- | --- | --- | *** | --- | --- | --- | |
| CL 177 | *** | *** | *** | *** | *** | *** | ** | --- | |
| CL 187 | *** | *** | --- | --- | --- | --- | --- | --- | |
| CL 189 | ** | *** | ** | --- | --- | --- | --- | --- | |
| CL 227 | *** | *** | ** | ** | ** | --- | --- | --- | |
| CL 251 | --- | --- | --- | *** | *** | --- | --- | --- | |
| CL 275 | --- | --- | --- | --- | *** | --- | --- | --- | |
| CL 4 | ** | *** | ** | ** | ** | *** | *** | *L. perenne* middle repetitive unit (AF063226) | |
| CL 59 | *** | *** | *** | *** | *** | *** | *** | *L. perenne* CACTA family | |
| CL 67 | *** | *** | *** | *** | *** | *** | *** | FaH13 *arundinacea* repeat element (AJ310138) | |
| CL 80 | ** | ** | ** | ** | ** | *** | *** | *L. perenne* rk1 gene (AM489608) | |
| CL 182 | *** | *** | *** | *** | *** | --- | --- | Pc68LrkB6 repetitive DNA (AY038008) | |

**^1^** Number of cluster obtained from comparative analysis

**^2^** Presence of tandem repeats in the genome based on *in silico* analysis

* Different number of asterix correspond to abundancy of individual tandem repeat within analyzed genomes (based on *in silico* analysis)
