# Supplementary figures and images for: Comparative analyses of DNA repeats and identification of a novel Fesreba centromeric element in fescues and ryegrasses
Source: BMC Plant Biol. 2020 Jun 17;20:280. doi: 10.1186/s12870-020-02495-0 (PMC7302162; doi:10.1186/s12870-020-02495-0)

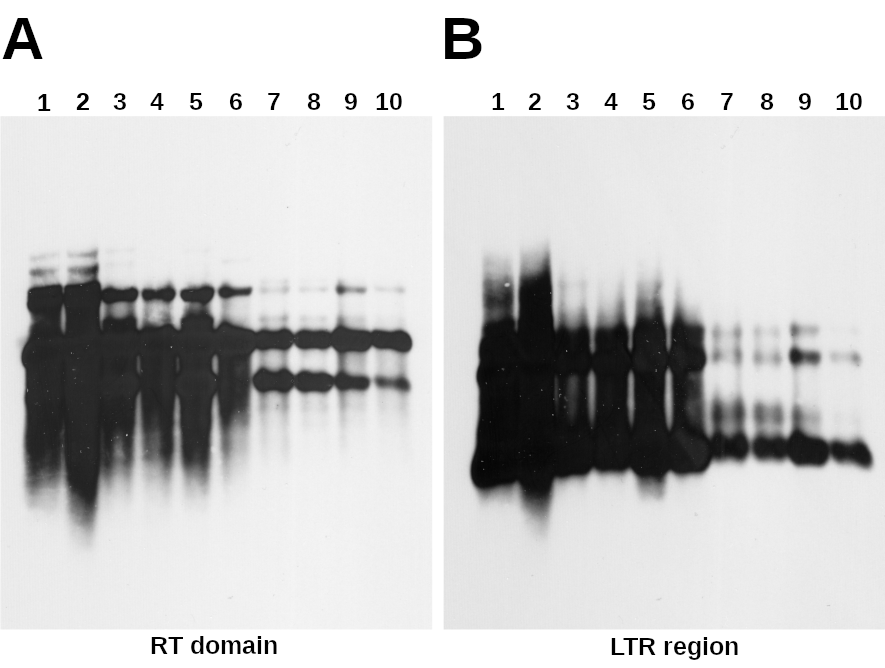

Supplement: Supplementary file 2 — Additional file 2: Fig. S1. Southern blots for the RT domain and non-coding LTR part of the Fesreba element. Southern blots were made with probes for the reverse transcriptase domain (A) and non-coding LTR region (B) of the Fesreba element. Lanes contained genomic DNA digested by HaeIII restriction endonuclease. Lane 1: diploid F. pratensis cv. Fure; lane 2: tetraploid F. pratensis cv. Westa; lane 3: hexaploid F. arundinacea subsp. arundinacea; lane 4: hexaploid F. gigantea; lane 5: tetraploid F. glaucescens; lane 6: tetraploid F. mairei; lane 7: tetraploid L. multiflorum cv. Mitos; lane 8: diploid L. multiflorum cv. Kuri1; lane 9: tetraploid L. perenne cv. Neptun; lane 10: diploid L. perenne. [file 12870_2020_2495_MOESM2_ESM.tiff]

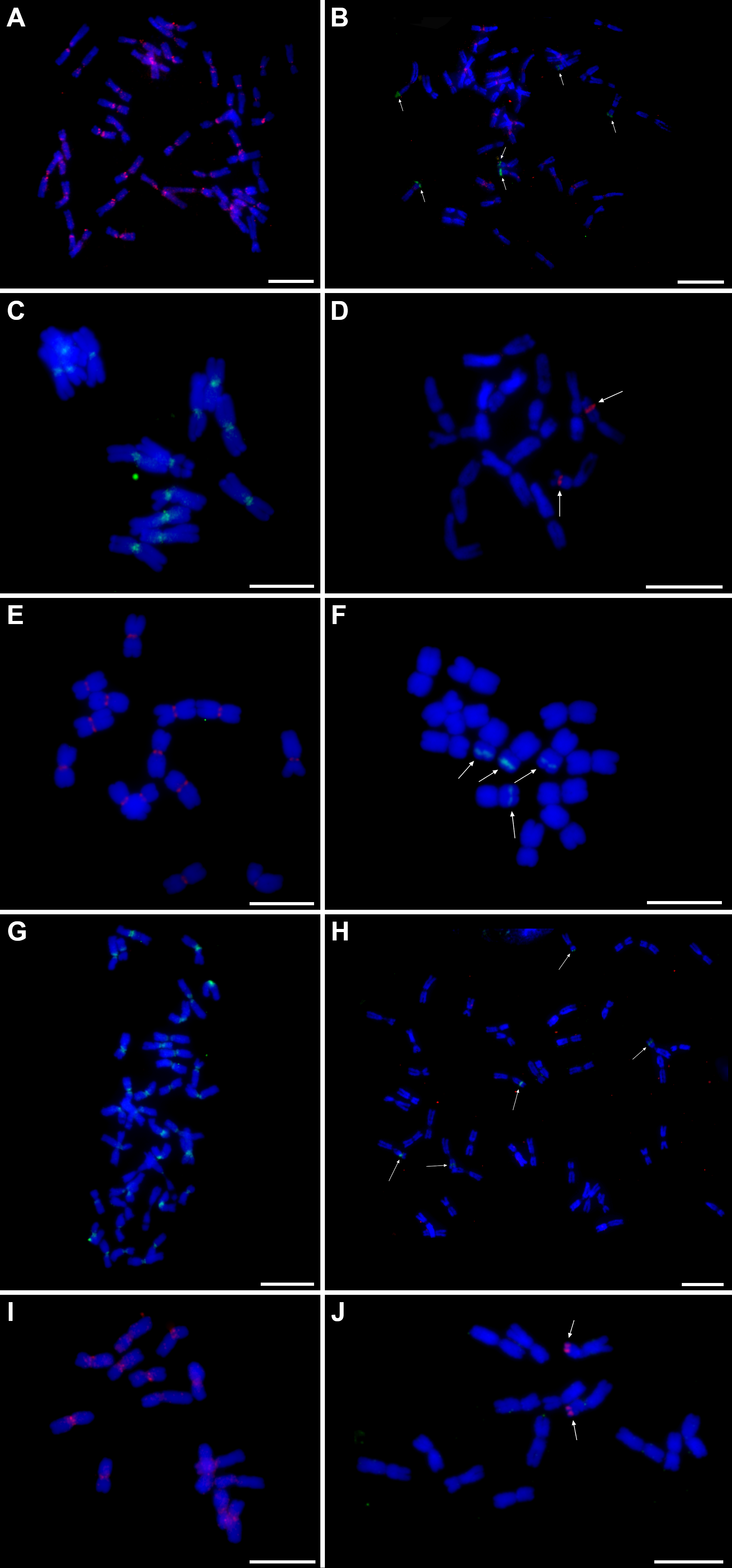

Supplement: Supplementary file 4 — Additional file 4: Fig. S2. Localization of the centromeric LTR retrotransposon Fesreba on mitotic chromosomes with fluorescence in situ hybridization. Mitotic metaphase plates were hybridized with a probe for the reverse transcriptase domain of the Fesreba element (A, C, E, G, I) and with a combination of probes for the non-coding LTR part of the Fesreba element and 45S rDNA, which served as control (B, D, F, H, J). (A, B) Avena sativa cv. Atego (2n = 2x = 14). (C, D) Secale cereale cv. Dánkowskie Diament (2n = 2x = 14). (E, F) Hordeum vulgare cv. Morex (2n = 2x = 14). (G, H) Triticum aestivum cv. Chinese Spring (2n = 6x = 42). (I, J) Aegilops tauschii (2n = 2x = 14). Signals corresponding to 45S rDNA loci are marked by arrows. Hybridization signals of a probe for the LTR region of the Fesreba element were absent in all related species (D, F, H, J) except A. sativa (B). Chromosomes were counterstained with DAPI (blue). The bar corresponds to 10 μm. [file 12870_2020_2495_MOESM4_ESM.tif]

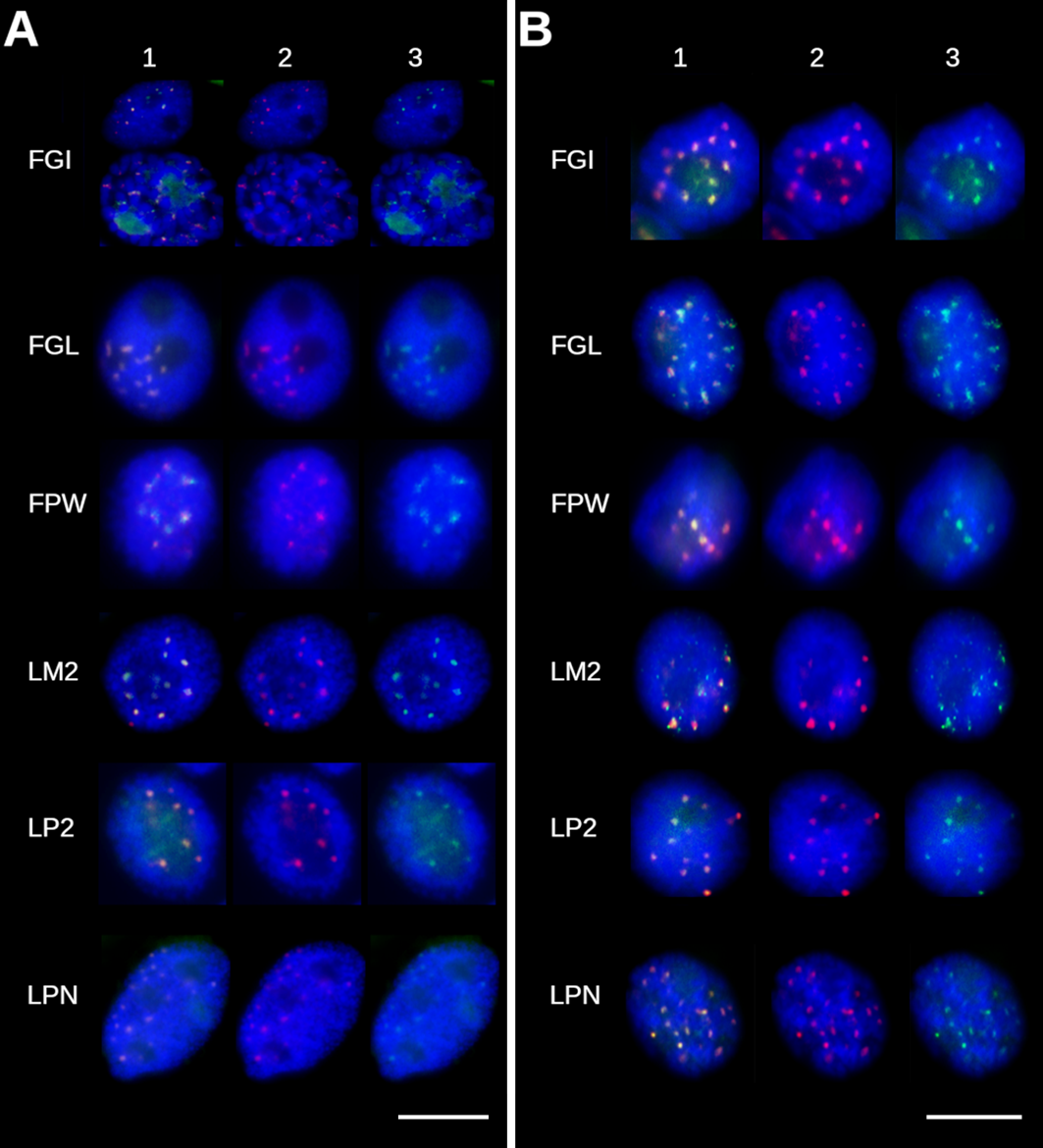

Supplement: Supplementary file 5 — Additional file 5: Fig. S3. Co-localization of CENH3 with the Fesreba element in three Festuca and three Lolium species. Immunolocalization of the histone H3 variant CENH3 (red) and FISH with probes for the reverse transcriptase (RT) domain and non-coding LTR part of the Fesreba element (green). F. gigantea (FGI); F. glaucescens (FGL); F. pratensis Westa (FPW); L. multiflorum Lm2 (LM2); L. perenne Neptun (LP2); L. perenne (LPN). Column 1 shows merged images, column 2 shows CENH3 signals (red), and column 3 shows FISH signals corresponding to the Fesreba element. In all accessions, the signals of CENH3 and FISH probes are overlapping. Nuclei were counterstained with DAPI (blue). The bar corresponds to 10 μm. [file 12870_2020_2495_MOESM5_ESM.tif]

**A**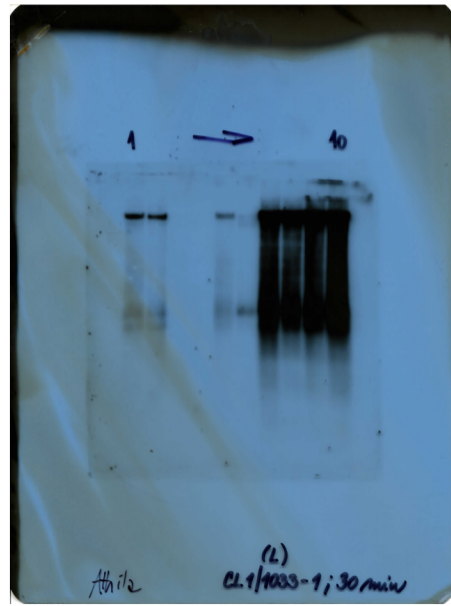**B**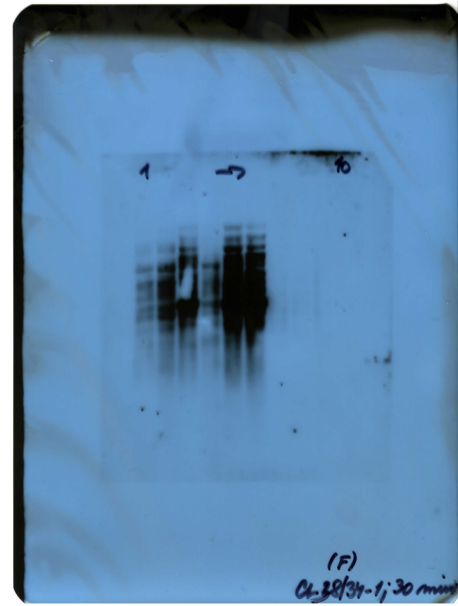**C**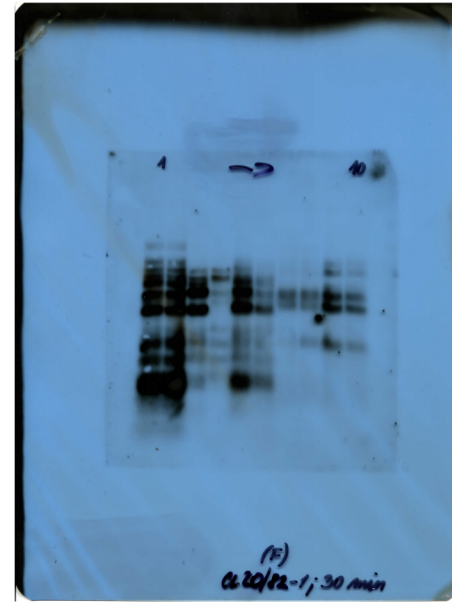**D**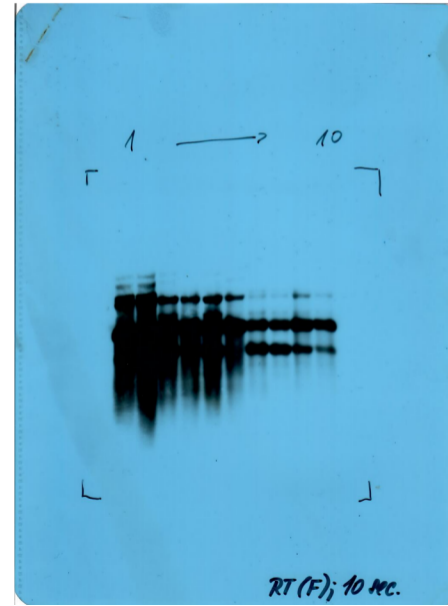**E**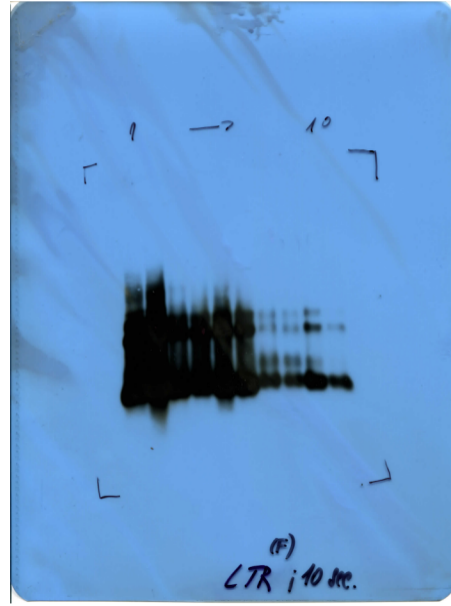

Supplement: Supplementary file 7 — Additional file 7: Fig. S4. Original images of Southern hybridization depicted in Fig. 5 and Additional file 2: Fig. S1, respectively. Original images of Southern hybridization with sequences derived from cluster CL1 (A), cluster CL38 (B), and cluster CL20 (C) and with sequences for the reverse transcriptase domain (D) and non-coding LTR region (E) of the Fesreba element. Lanes contained genomic DNA digested by HaeIII restriction endonuclease. Lane 1: diploid F. pratensis cv. Fure; lane 2: tetraploid F. pratensis cv. Westa; lane 3: hexaploid F. arundinacea subsp. arundinacea; lane 4: hexaploid F. gigantea; lane 5: tetraploid F. glaucescens; lane 6: tetraploid F. mairei; lane 7: tetraploid L. multiflorum cv. Mitos; lane 8: diploid L. multiflorum cv. Kuri1; lane 9: tetraploid L. perenne cv. Neptun; lane 10: diploid L. perenne. [file 12870_2020_2495_MOESM7_ESM.pdf]
