# Supplementary material for: Comparative analyses of DNA repeats and identification of a novel Fesreba centromeric element in fescues and ryegrasses
Source: BMC Plant Biol. 2020 Jun 17;20:280. doi: 10.1186/s12870-020-02495-0 (PMC7302162; doi:10.1186/s12870-020-02495-0)
Supplement: Supplementary file 3 — Additional file 3: Table S2. Representation of the RT domain and non-coding part of the LTR region of the Fesreba element estimated by ddPCR. Copy numbers of the reverse transcriptase (RT) domain and non-coding part of the LTR region of the Fesreba element were estimated with droplet digital PCR. Values are averages of three independent experiments with standard deviations. [file 12870_2020_2495_MOESM3_ESM.docx]

**Table S2.** Representation of RT domain and non-coding part of LTR region of the Fesreba estimated by ddPCR.

| **accession** | **enzyme** | **copies/monoploid genome (1Cx)** | | | |
| --- | --- | --- | --- | --- | --- |
|  |  | **RT domain** | **± SD** | **Non-coding LTR** | **± SD** |
| *Festuca pratensis* Huds. cv. Fure | *Hpa*I | 847 | 59 | 4402 | 133 |
|  | *Hpa*II | 972 | 22 | 5102 | 133 |
| *Festuca pratensis* Huds. cv. Westa | *Hpa*I | 2147 | 79 | 3577 | 96 |
|  | *Hpa*II | 2720 | 68 | 3883 | 263 |
| *Festuca arundinacea* Schreb. ssp. *arundinacea* | *Hpa*I | 139 | 6 | 2873 | 88 |
|  | *Hpa*II | 136 | 8 | 4009 | 88 |
| *Festuca gigantea* L. GR11759 | *Hpa*I | 272 | 12 | 3401 | 184 |
|  | *Hpa*II | 339 | 14 | 4595 | 180 |
| *Festuca* Schreb. ssp. *glaucescens* | *Hpa*I | 77 | 7 | 4041 | 85 |
|  | *Hpa*II | 165 | 21 | 6930 | 147 |
| *Festuca mairei* GR610941 | *Hpa*I | 135 | 5 | 3367 | 166 |
|  | *Hpa*II | 207 | 15 | 3688 | 184 |
| *Lolium multiflorum* Lam. | *Hpa*I | 272 | 8 | 1979 | 49 |
|  | *Hpa*II | 384 | 7 | 1951 | 109 |
| *Lolium multiflorum* Lam. cv. Mitos | *Hpa*I | 320 | 10 | 2115 | 38 |
|  | *Hpa*II | 202 | 8 | 2023 | 132 |
| *Lolium perenne* L. GR3320 | *Hpa*I | 174 | 4 | 1650 | 95 |
|  | *Hpa*II | 158 | 5 | 1645 | 42 |
| *Lolium perenne* L. cv. Neptun | *Hpa*I | 173 | 4 | 1554 | 20 |
|  | *Hpa*II | 112 | 3 | 1563 | 28 |

Copy number estimation of reverse transcriptase (RT) domain and non-coding part of LTR region of Fesreba element done by droplet digital PCR. Values are averages of three independent experiments with standard deviation.
