# Supplementary material for: Comparative analyses of DNA repeats and identification of a novel Fesreba centromeric element in fescues and ryegrasses
Source: BMC Plant Biol. 2020 Jun 17;20:280. doi: 10.1186/s12870-020-02495-0 (PMC7302162; doi:10.1186/s12870-020-02495-0)
Supplement: Supplementary file 6 — Additional file 6: Table S3. Primers used for PCR amplification of DNA repeats. [file 12870_2020_2495_MOESM6_ESM.docx]

**Table S3.** Primers used for PCR amplification of DNA repeats.

| **DNA element** | **Primer name** | **Primer sequence** |
| --- | --- | --- |
| *Ty3/Gypsy* Athila  CL1/173 | CL_1/173_L | 5´-GGGGTGTGGAGTTTTCTCAA |
|  | CL_1/173_R | 5´-TGCTTTAGTTCGCATGTTGG |
| *Ty3/Gypsy* Athila  CL1/237 | CL_1/237_L | 5´-AAATTTGGCTTTCACGCAAC |
|  | CL_1/237_R | 5´-TTTTCAATGGTGGCAAGTCA |
| *Ty3/Gypsy* Athila  CL1/373 | CL_1/373_L | 5´-TATTGGGTTGACTTGGCACA |
|  | CL_1/373_R | 5´-TGCCATCAAGTTCGTGAGAG |
| *Ty3/Gypsy* Athila  CL1/376 | CL_1/376_L | 5´-CCAAGCTTGAGCTTTTGTCC |
|  | CL_1/376_R | 5´-GAGCGGAATTTTGAGCTGTC |
| *Ty3/Gypsy* Athila  CL1/574 | CL_1/574_L | 5´-AGATGACGAAGGAAGCCAGA |
|  | CL_1/574_R | 5´-ACCTTGGTTCAATGGTTTCG |
| *Ty3/Gypsy* Athila  CL1/1033 | CL_1/1033_L | 5´-AACCCAGCATGACCAAGAAC |
|  | CL_1/1033_R | 5´-ATTGCTTGCGAAACCTTGAT |
| *Ty3/Gypsy* Athila  CL1/1093 | CL_1/1093_L | 5´-ACCAGGAAGCTCATGAATGG |
|  | CL_1/1093_R | 5´-GGGAGGACATTGTGCCTTTA |
| *Ty3/Gypsy* Athila  CL38/34 | CL_38/34_L | 5´-CCTTGGTCATGCTGGATTTT |
|  | CL_38/34_R | 5´-TCCACCGAATCATTCTAGGC |
| *Ty3/Gypsy* Athila  CL49/96 | CL_49/96_L | 5´-GGTTTCTCCTGCAGCTATGC |
|  | CL_49/96_R | 5´-TCCTTGTCTCTTTGCCTCGT |
| *Ty3/Gypsy* Chromoviridae  CL16/93 | CL_16/93_L | 5´-GAAGACACAAGGGCAACCAT |
|  | CL_16/93_R | 5´-TGAGGAGCGAGTTCACCTTT |
| *Ty3/Gypsy* Chromoviridae  CL34/230 | CL_34/230_L | 5´-CACCTTCGGTTCAGCTCTTC |
|  | CL_34/230_R | 5´-CTTTGCGGATTTGATTTGGT |
| *Ty3/Gypsy* Chromoviridae  CL36/176 | CL_36/176_L | 5´-CCAAAGGCTGCTGATACCAT |
|  | CL_36/176_R | 5´-TGGGACCCCTCCTTTACTCT |
| *Ty3/Gypsy* Ogre-Tat CL8/209 | CL_8/209_L | 5´-GTCGACTTCGTCAACTGCAA |
|  | CL_8/209_R | 5´-CAGGATCGACGTACGGATTT |
| *Ty3/Gypsy* Ogre-Tat CL8/394 | CL_8/394_L | 5´-CTCTCCCGACGCAAAAGTAG |
|  | CL_8/394_R | 5´-CGGGTCGAACTCAGAGAGTC |
| *Ty3/Gypsy* Ogre-Tat CL11/13 | CL_11/13_L | 5´-TTGCGGGAGCTGTACTCTT |
|  | CL_11/13_R | 5´-CTCGAAGTTCCGATCTCGTC |
| *Ty3/Gypsy* Ogre-Tat CL14/268 | CL_14/268_L | 5´-TTGAGGCACAAAATGCAGAG |
|  | CL_14/268_R | 5´-GTCTGGCAGGCATCTTTCTC |
| *Ty3/Gypsy* Ogre-Tat CL18/82 | CL_18/82_L | 5´-CACACCCACCAAGTCATCAG |
|  | CL_18/82_R | 5´-ATAATTGCCGCGTTGAAAAC |
| *Ty3/Gypsy* Ogre-Tat CL20/64 | CL_20/64_L | 5´-AGTTCCCCACCACAGTCAAG |
|  | CL_20/64_R | 5´-TCACTGAGCCAACAGACAGG |
| LTR unclassified CL12/134 | CL_12/134_L | 5´-TGCGGTGAAAACCCTAAATC |
|  | CL_12/134_R | 5´-TGGAGATGCCTCGCTTAGTT |
| LTR unclassified CL25/212 | CL_25/212_L | 5´-CCGTTATGGTTGCTCCTTGT |
|  | CL_25/212_R | 5´-GCAATGCTCTTCTGGTCACA |
| LTR unclassified  CL74/39 | CL_74/39_L | 5´-CGGCTCAGAAAAGAACCTTG |
|  | CL_74/39_R | 5´-AACGAACCCAGCAAATTGTC |
| LTR unclassified  CL74/57 | CL_74/57_L | 5´-GGGAATCGTCCAAGTCTTCA |
|  | CL_74/57_R | 5´-TTGCTGACAGCCAACAAGAC |
| DNA repeat unclassified  CL27/109 | CL_27/109_L | 5´-GGGTATCCAACGTGCTCTGT |
|  | CL_27/109_R | 5´-AGCTCTCCTTCTTCCGGTTC |
| Fesreba_RT | CL38_RT_L | 5´-GGCGCATGTGTGTAGATTGT |
|  | CL38_RT_R | 5´-ATCACGCAACACTTGCAAAA |
| Fesreba_4F/CL38/LTR | CL_38/LTR_L | 5´-TTCTGGGTCGGTTATTTTCG |
|  | CL_38/LTR_R | 5´-GATTGATTCCTGTCGCCAAT |
